# Research and Evaluation in a Child-Focused Place-Based Initiative: West Philly Promise Neighborhood

**DOI:** 10.3390/ijerph20095716

**Published:** 2023-05-04

**Authors:** Félice Lê-Scherban, Irene Headen, Adena M. Klem, Kelley Traister, Erikka Gilliam, Maggie Beverly, Matthew Jannetti, Joanne Ferroni, Amy Carroll-Scott

**Affiliations:** 1 Department of Epidemiology and Biostatistics, Dornsife School of Public Health, Drexel University, Philadelphia, PA 19104, USA; 2 Drexel Urban Health Collaborative, Dornsife School of Public Health, Drexel University, Philadelphia, PA 19104, USA; 3 Department of Community Health and Prevention, Dornsife School of Public Health, Drexel University, Philadelphia, PA 19104, USA; 4 External Process Evaluator, Consultant to Office of University & Community Partnerships, Drexel University, Philadelphia, PA 19104, USA; 5 Office of University & Community Partnerships, Drexel University, Philadelphia, PA 19104, USA

**Keywords:** place-based intervention, children, community-engaged research, Promise Neighborhood, program evaluation, data integration, multisectoral, systems thinking

## Abstract

Place-based initiatives attempt to reduce persistent health inequities through multisectoral, cross-system collaborations incorporating multiple interventions targeted at varying levels from individuals to systems. Evaluations of these initiatives may be thought of as part of the community change process itself with a focus on real-time learning and accountability. We described the design, implementation, challenges, and initial results of an evaluation of the West Philly Promise Neighborhood, which is a comprehensive, child-focused place-based initiative in Philadelphia, Pennsylvania. Priorities for the evaluation were to build processes for and a culture of ongoing data collection, monitoring, and communication, with a focus on transparency, accountability, and data democratization; establish systems to collect data at multiple levels, with a focus on multiple uses of the data and future sustainability; and adhere to grant requirements on data collection and reporting. Data collection activities included the compilation of neighborhood-level indicators; the implementation of a program-tracking system; administrative data linkage; and neighborhood, school, and organizational surveys. Baseline results pointed to existing strengths in the neighborhood, such as the overwhelming majority of caregivers reporting that they read to their young children (86.9%), while other indicators showed areas of need for additional supports and were programmatic focuses for the initiative (e.g., about one-quarter of young children were not engaged in an early childhood education setting). Results were communicated in multiple formats. Challenges included aligning timelines, the measurement of relationship-building and other process-focused outcomes, data and technology limitations, and administrative and legal barriers. Evaluation approaches and funding models that acknowledge the importance of capacity-building processes and allow the development and measurement of population-level outcomes in a realistic timeframe are critical for measuring the success of place-based approaches.

## 1. Introduction

Persistent health inequities among the same populations and in the same geographic areas stem from deeply rooted historical and ongoing inequitable systems and policies across multiple domains (e.g., housing, health care, education, employment). Policies and practices in the United States (US) rooted in systemic racism shaped the clustering of populations of color into urban neighborhoods characterized by generations of disinvestment in the systems meant to serve those communities while simultaneously prioritizing investments into systems serving white, suburban communities [1]. For example, residential mortgage security maps created by the federally sponsored Home Owners’ Loan Corporation (HOLC) in the 1930s labeled majority Black areas as “hazardous”, effectively blocking Black residents from accessing mortgage financing and home ownership. To this day, residence in one of these so-called “historically redlined” neighborhoods—named because the maps depicted these neighborhoods with a red outline—is associated with poorer health [2]. Reducing these inequities therefore requires a multilevel, population-centered approach that simultaneously addresses these multiple systems affecting individuals’ health as well as the interactions between them. Place-based initiatives attempt to achieve this through multisectoral, cross-system collaborations incorporating multiple different interventions targeted at varying levels from individuals (e.g., health behavior and education programs) to systems (e.g., collective efficacy and investment efforts).

Increasing interest over the past several decades in the potential for place-based approaches to improve health equity [3] has been accompanied by increasing need for effective strategies to evaluate these initiatives. Reviews conducted over the past 15 years have identified common considerations, strategies, and challenges in evaluating place-based initiatives [4,5,6,7,8,9,10]. Most fundamentally, because of their inherently comprehensive nature and population-centered focus—with multiple overlapping interventions at multiple levels—traditional program evaluation methods may not be feasible and in fact may fail to capture change that is achieved between programs or services or at the systems level [9]. Instead, place-based initiatives can be thought of as complex adaptive systems [11], and their evaluation may be thought of as part of the community change process itself with a focus on real-time learning and accountability [4,6,12]. In keeping with this complexity, evaluations of place-based initiatives have incorporated models and approaches emphasizing participation, iteration, and adaptability such as developmental evaluation, realist evaluation, action research, quality improvement, and performance management [5,9,12]. Quantitative evaluation of outcomes tends to be based on time-trend or quasi-experimental designs, as randomized control trials are generally infeasible (except in some circumstances for limited-scope evaluation of specific interventions implemented within the context of broader place-based initiatives) [5,7,8].

Unsurprisingly, commonly reported challenges in evaluating place-based initiatives also reflect their inherently complex nature. Place-based initiatives often have comprehensive objectives but lack a clear theory of change, or they have multiple or changing underlying theories of change [4,7,10,13]. There can also be conflicting approaches to the evaluation itself, including philosophies that favor more “objective” or more “experience-based” approaches [6,7] or tension in multi-site programs between the overall evaluation and local within-site evaluations [13]. Place-based initiatives are also subject to midstream changes in objectives or strategies stemming from internal evolution of the initiative or external forces such as changes in funding, policy, or oversight [6,8,13]. Defining and identifying the relevant population can be difficult because of residential mobility or misalignment of the initiative’s geographic footprint with administrative boundaries [5,7,8,13]. Common measurement challenges include difficulty measuring systems-level change and collaboration—often key goals of place-based initiatives, lack of a standard intervention and difficulty in measuring “dose” of intervention, lack of a control population, and logistical challenges in collecting data [5,6,7]. When change in outcomes is detected, attribution to the initiative is complicated by underlying changes in the community and concurrent, non-initiative-related policies and interventions [6,7,13]. In addition to the attribution of outcomes to the initiative overall, the attribution of change to specific programs or organizations within the initiative is also challenging and may be politically sensitive [6]. Finally, timeline is perhaps the most consistently identified challenge: achieving measurable, population-level change in outcomes, particularly in the context of the persistent structurally-driven social inequities place-based initiatives aim to address, is a long-term process that requires considerably longer than a typical funding cycle.

Evaluations of place-based initiatives tend to produce information that is useful for initiative implementation and funder reporting but not always aligned with academic publishing norms. Resultshave been largely reported in the gray literature or in education and social-service disciplines [12,13]. The peer-reviewed public health literature therefore lacks studies describing evaluation of comprehensive place-based initiatives despite the relevance—and often explicit focus—of these initiatives on improving health. We address this gap by describing the design, implementation, and initial results of an evaluation of the West Philly Promise Neighborhood (WPPN), which is a comprehensive, child-focused, place-based initiative in Philadelphia, Pennsylvania. We also discuss how common challenges in evaluating place-based initiatives identified in the literature manifest in the WPPN evaluation.

## 2. Materials and Methods

### 2.1. Setting

WPPN is a place-based initiative to provide cradle-to-career supports for children living or attending school in a two-square-mile section of Philadelphia as well as their families and communities. The overarching goal of the initiative is to improve children’s long-term educational, health, economic, and developmental outcomes. WPPN was funded through a $30 million grant from 2017 to 2021 (extended through June 2023 in a no-cost extension as a result of the COVID-19 pandemic) as part of the US Department of Education’s Promise Neighborhoods program. The Promise Neighborhoods program is based on the Harlem Children’s Zone model and funds place-based initiatives in over 50 urban and rural communities throughout the US [3,14,15]. Like other place-based initiatives, the Promise Neighborhoods program employs a saturation model in which multiple complementary services are implemented within the same geographic footprint [5]. Although there is a focus on schools and educational outcomes, the goals of the program are comprehensive. Grantees are required to set targets for and report on population-level indicators of outcomes ranging from academic performance and student attendance to health care access, diet, physical activity, and community safety [16]. Of note, the grant program also has a capacity- and systems-building emphasis as opposed to focusing solely on the implementation of individual programs.

WPPN therefore comprises an array of programmatic, capacity-building, and systems coordination activities, including both school-based and community-based activities. Drexel University (“Drexel”) serves as the lead agency with over twenty community, governmental, service-provision, and civic partner organizations providing supportive activities. The initiative includes seven partner schools: six public schools (one high school, one middle school, one kindergarten-grade 5, two kindergarten-grade 8) and one kindergarten-grade 12 charter school. WPPN’s geographic footprint was designed to be coterminous with the West Philadelphia Promise Zone, which is a US Department of Housing and Urban Development designation to promote economic development in areas with high poverty but also high assets [17]. The Promise Zone and Promise Neighborhood initiatives are distinct, despite their similar names, resulting from concurrent national place-based initiative strategies implemented during the Obama presidential administration. However, the Promise Zone designation confers priority points for US governmental funding that helped secure WPPN funding, and WPPN employs a collective impact approach that draws from the existing partnerships and committee structure developed as part of the West Philadelphia Promise Zone [18].

The WPPN leadership structure within Drexel includes a Principal Investigator and Director, both of whom are housed in Drexel’s Office of University and Community Partnerships along with WPPN outreach, communication, and management staff who together make up the Implementation Team. Faculty members from Drexel’s School of Education and School of Public Health serve as Co-Principal Investigators. The WPPN coalition includes a multisectoral, multilevel Project Management Team charged with managing initiative-wide coordination and prioritization. The Project Management Team is led by the Director and includes representatives from WPPN-funded programmatic partners, other programmatic and service-provision partner organizations serving children and families in West Philadelphia, and public agencies. A cross-neighborhood Community Advisory Council advises on program implementation and reach, accountability of the initiative to the community, and communication. The Community Advisory Council includes representatives from neighborhood civic councils, community development corporations, faith organizations, and WPPN partner schools. The initiative’s research and evaluation activities are led by a Data and Research Core, which is housed at the Drexel Urban Health Collaborative at the School of Public Health [19].

### 2.2. Target Population

The WPPN cohort of children is an open cohort defined as any child or young adult who at any time during the original grant period (calendar years 2017–2021) was between the ages of 0 and 18 and resided in the WPPN geographic footprint and/or attended one of the initiative’s partner schools. If a child was included in the cohort in any year and was above the age of 18 in any subsequent year, we still attempted to collect follow-up data related to them for the duration of the grant.

As with other place-based initiatives, identifying the WPPN population was complicated by high residential and student mobility in West Philadelphia [20]. Specifics of the WPPN cohort definition created additional challenges. There is no existing data source to enumerate the population of children and their families living in the WPPN geographic footprint. In addition, at any given point in time, the large majority of school-aged children either attend a WPPN partner school and do not reside within the WPPN footprint or live in the footprint and do not attend a WPPN school. This is because there are additional public and private schools in the WPPN footprint that are not WPPN partner schools, and because Philadelphia has both a large charter school presence and a strong school choice program through which students can apply to attend schools outside their zoned catchment school using a centralized application, lottery, and waitlist system [21]. As a result, many Philadelphia students attend schools outside of their residential neighborhoods. Furthermore, some cohort members who reside in the WPPN footprint are too young to attend school or are aged 18 or younger but no longer students. To address these cohort definition challenges, we triangulated information from the US Census, our neighborhood survey sampling frame (described below), and neighborhood survey responses to estimate the size and characteristics of our cohort.

### 2.3. Outcomes

Table 1 lists the grant-required indicators and their sources. The specific measures and sources are defined by the Promise Neighborhoods program and are required to be consistent across local grantees. In keeping with grant guidance [16], indicator data came from the neighborhood survey, school survey, and school district administrative records. Of note, although all indicators are measured at a population level (as opposed to only among participants in specific WPPN activities), different indicators pertain to different subpopulations of the WPPN child cohort. In some cases, this was by design: for example, indicator 3 (childcare) pertains only to children aged <5 years. In other cases, this was a function of the data source: for example, information for indicator 1 (medical home) was collected in the neighborhood survey and therefore includes only children living in the WPPN footprint.

### 2.4. Evaluation Framework and Design

**Figure 1:**
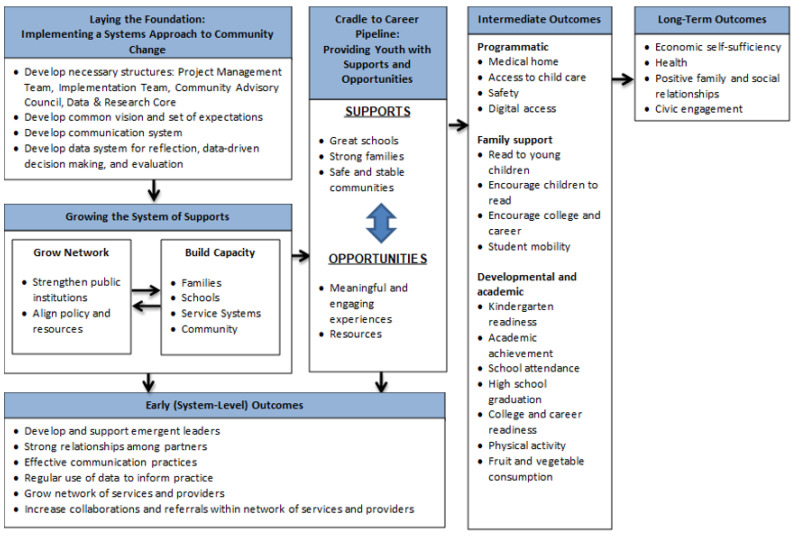
West Philly Promise Neighborhood theory of change.

The WPPN theory of change (Figure 1) was developed in early 2018, incorporating input on an early draft from community stakeholders collected during a community meeting. The theory of change aligned with the desired systems-level outcomes and the 15 grant-mandated indicators such that the early outcomes capture systems-level change while the intermediate programmatic, family supports, and child outcomes (developmental and academic) capture change in the grant indicators. We theorized that improvements in these intermediate outcomes will, in turn, lead to improvements in longer-term outcomes for children as they transition to adulthood.

**Figure 2:**
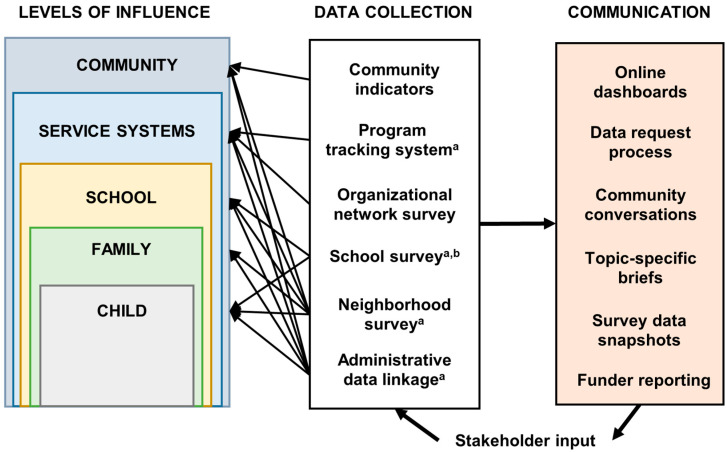
Evaluation approach. Data collection methods were designed to capture information at each of the relevant levels of influence. For example, community-level data were collected through community indicators, a population-based neighborhood resident survey, and administrative data linkage. Some data collection methods also provided information about multiple different levels of influence. Stakeholder input based on communication of data and research results is incorporated into future data collection and communication activities. ^a^ Grant requirement. ^b^ School survey, and associated online data reporting and requests, developed and maintained by School District.

In addition to being structured around the 15 grant-mandated indicators, both the WPPN theory of change and associated evaluation were informed by the socioecological model, which emphasizes the multilevel contexts and interactions between these contexts that influence individuals’ health [22,23]. A core proposition of the socioecological model is that multilevel interventions at the individual-, community-, and policy-level are necessary for achieving significant positive changes. In the context of WPPN, we identified relevant levels influencing child health and development: individual, family, school, service systems, and community (Figure 2). It was therefore important to incorporate information at these different levels into the evaluation.

There were several major considerations in planning research and evaluation activities for WPPN. First, the Promise Neighborhoods grant mechanism mandated several data collection activities including a neighborhood survey, school survey, program participation system, and a data system designed to integrate these data [16,24]. While these activities were mandatory, their specific design and implementation were allowed to be tailored to the needs of the Promise Neighborhood grantee. We describe our approach to these activities, as well as how they related to collection of mandatory reporting indicators, below.

Second, establishing participatory, transparent processes for data collection, reporting, and use were of paramount importance. WPPN-related evaluation activities could increase an already unsustainable amount of duplicative and disruptive research in these neighborhoods, which are comprised of a majority of African American residents who experience dramatically higher poverty and unemployment rates and lower median household income relative to other Philadelphia neighborhoods [20]. Residents in West Philadelphia have largely not benefited from the economic growth of its surrounding “eds and meds” such as Drexel University, the University of Pennsylvania, the Children’s Hospital of Philadelphia, and the Hospital of the University of Pennsylvania. Residents have voiced concerns about research coercion, fatigue, the need for tangible benefits of research to their community, and a desire to partner in the research process to help conceptualize the research that would best inform community-led efforts [25]. These concerns occur in the context of a long-established and well-known history of ethical abuses in research among socioeconomically disadvantaged and minority populations that causes mistrust of researchers, city systems, and the data they produce [26,27,28,29].

The third consideration was timeline: as has been previously noted in the context of place-based initiatives [13,30], observing large-scale, detectable, population-level change within the 5-year grant duration would be unlikely. Rather, we prioritized establishing ongoing processes for data collection, monitoring, and reporting that would allow not only for the detection of changes over time but also for the concurrent use of the data for multiple purposes by stakeholders including program planning and advocacy, and the establishment of a data infrastructure to support other programs and place-based initiatives occurring in the WPPN/Promise Zone footprint.

Given these considerations, we identified the following three priorities for the WPPN evaluation:

1. Ensure all data and research activities adhere to guiding principles of transparency, accountability, and data democratization.
2. Establish systems to collect, monitor, and communicate data at multiple levels with focus on multiple uses of the data and future sustainability.
3. Adhere to grant requirements on data collection and reporting.

Figure 2 summarizes research and evaluation activities based on these priorities. Data collection activities included those mandated by the grant as well as additional activities to meet the priorities of the evaluation. Data collection methods were designed to capture information at each of the relevant levels of influence. For example, community-level data were collected through community indicators (e.g., census-tract-level median income), a population-based neighborhood resident survey (e.g., perceived social cohesion), and administrative data linkage (e.g., census-tract-level behavioral health service utilization rate). Some data collection methods also provided information about multiple different levels of influence.

We compiled community indicator data from a variety of publicly available sources including US Census data and OpenDataPhilly, Philadelphia’s online public data portal [31]. Indicators were chosen on a variety of topics based on priorities of the WPPN initiative as well as additional priorities identified by community partners over the course of the initiative and included demographics, education, housing, and public safety [32]. Indicators were aggregated to the census tract level to provide localized information for community programming and advocacy efforts; the WPPN footprint includes all or portions of 15 census tracts. We also created indicators aggregated to the entire WPPN footprint. A challenge in doing this was that the footprint boundaries were created to align with social and economic priorities (for example, the footprint coincides with a US Department of Housing and Urban Development-designated Promise Zone [17]) but do not align with census tract boundaries. Because of highly variable land-use patterns within the footprint, which contains retail, university campus, student housing, rail and transit areas in addition to family residential areas, weighting census tracts by the proportion of land area falling within the footprint would not produce accurate estimates for our population of interest. Therefore, for census tracts falling partially within the footprint, we created weights based on the proportion of the population of families with children residing within the footprint based on census block-level data.

We developed and implemented a program tracking system, called the Promise Information Portal (PIP), to capture and monitor information about child, family, and school and service staff participation in WPPN-funded activities. In addition to program participation information, the PIP houses a detailed inventory of services and resources available in the WPPN footprint that were vetted through environmental scan methods and community resident ground truthing. It is also used by WPPN staff to house program performance indicator information for funder reporting. The PIP was developed by a contracted vendor with previous experience working with Promise Neighborhood grantees and is maintained by a WPPN staff system administrator. Because the primary users of the system would be WPPN partner program providers, it was intentionally created to be highly customizable in order to accommodate data and reporting needs for different types of organizations, including reducing duplicative data entry. For programs entering individual-level participation data into the PIP, informed consent was obtained from participants.

Given the initiative’s focus on systems building, we captured information about communication and collaboration between WPPN partners through conducting an organizational network survey of partner organizations. Leveraging network analysis methods, we obtained information on each organization’s interactions with all other WPPN partner organizations in the areas of resource sharing, strategic collaboration (e.g., co-participation on task forces, joint fundraising), care coordination, referrals, and data sharing.

One of the grant-mandated data collection activities was a school survey of students in WPPN partner schools. However, the School District of Philadelphia (“District”) conducts an ongoing annual survey of students [33]. Rather than overburden schools and students with an additional survey, we used this existing survey as the WPPN school survey and collaborated with the District to ensure questions necessary for the evaluation were included.

The grant also mandated a neighborhood survey of families with children living in the WPPN footprint. Based on stakeholder input, we designed the WPPN neighborhood survey as a longitudinal cohort study of a representative sample of families with three waves of data collection over the course of the grant period. Participants were caregivers of children aged 0–18 at the time of recruitment; in waves 2 and 3, new participants were recruited in addition to recontact with existing participants. We collaborated with the District and the City of Philadelphia (“City”) to develop an address-based sampling frame using addresses associated with school enrollment and service utilization records [34]. Surveys were conducted through in-person outreach by community resident surveyors. The surveys included a variety of topics including child and caregiver health, education, community built and social environments, neighborhood resources, and awareness and perceptions of the WPPN initiative. In developing the survey, we first consulted with the WPPN Project Management Team and Community Advisory Council about important topic domains for inclusion. Draft survey questionnaires were then reviewed by stakeholders including the WPPN Project Management Team and Community Advisory Council, piloted with a group of caregivers from adjacent neighborhoods, and then revised according to their input.

Finally, we conducted administrative data linkage of individual-level data about the WPPN cohort to answer research and evaluation questions for the initiative. The initial intent was to establish an integrated data system (IDS) [35,36,37,38] with ongoing linkage of data from multiple sources (academic records, vital statistics and service utilization data, WPPN program participation data) to use for ongoing research, evaluation, and initiative performance improvement. However, because of administrative and legal barriers, we were unable to establish the IDS during the grant period and instead conducted two one-time linkages to answer limited-scope research questions. The first was a linkage of academic record, vital statistics, and public service utilization data to develop early-life risk profiles predictive of 3rd-grade school-related outcomes (standardized test performance, attendance) among a subcohort of WPPN students. The second, still ongoing, is a linkage of academic record and WPPN program participation data to evaluate associations of participation in various numbers and types of WPPN activities with school-related outcomes. This second linkage incorporates a quasi-experimental design in which WPPN cohort students will be matched with students from outside WPPN. It will also allow for the investigation of differential effects according to the “dose” of WPPN programming each child received.

All WPPN research activities including human subjects (neighborhood survey, administrative data linkages, organizational network survey) have been approved by the Drexel University Institutional Review Board. The WPPN neighborhood survey and administrative data linkages were additionally approved by the City of Philadelphia Institutional Review Board and School District of Philadelphia Research Review Board because of the inclusion of data from those agencies.

## 3. Results

### 3.1. Population

According to the American Community Survey (ACS) estimates, the WPPN resident cohort comprised 7765 children aged 0–17 years at baseline in 2017, representing 20% of the total population in the WPPN geographic footprint [20]. Approximately 71% of those children were school-aged (i.e., 5–17 years). Based on school information reported in the WPPN neighborhood survey, approximately 93% of students living in the WPPN footprint attended a public or charter school, while 7% attended a private school. Based on information provided by the District, among the subset of the WPPN student cohort who attended a public or charter school, 28% attended a WPPN partner school but did not live in the WPPN footprint, while 72% lived in the WPPN footprint.

### 3.2. WPPN Programming (Supports and Opportunities)

As in many place-based initiatives, while laying the foundation for a systems approach and increasing systems-level coordination were primary objectives, the majority of funding was directed toward individual programs for children and families. Programs were school- or community-based and selected to help WPPN schools and communities attain the intermediate outcomes in the theory of change (Figure 1) and aligned directly with the federally mandated outcome indicators. For example, programs to promote academic achievement included funding professional development opportunities for math and English/Language Arts teachers; math interventionists to support struggling students; and summer camps in literacy, science, and STEM (science, technology, engineering, and math). Programs to promote school climate, safety, and attendance included funding or leveraging city-wide funding opportunities to provide each partner school with an Out of School Time (OST) partner, funding a Professional Learning Community for OST providers to strengthen the effectiveness of these programs and encourage their use of best practices, and partnering with the City of Philadelphia to co-fund the transition of one of the WPPN schools into a community school, which is a public school that provides services and supports designed to fit the community’s needs [39]. Programs to support high school graduation and college and career awareness included funding college/career coaches in high schools to mentor high school students on college preparation, providing career-oriented workshops and work experiences, and coordinating tours of college campuses. 

Between the academic years 2017–2018 and 2021–2022, the WPPN funded a total of 62 unique programs or positions in the seven partner schools or the community. The number of programs ranged from 24 to 48 per year with many programs funded for multiple years. Table 2 shows the number of funded programs per year by focus area.

### 3.3. Outcomes

Mandated outcome reporting for the Promise Neighborhoods program uses a time-series approach in which data are collected and compared annually or at three time-points throughout the grant period, depending on the source.

Table 3 shows results over time for required indicators estimated using data from the three waves of the neighborhood survey. Results for indicators estimated using school district records and school surveys are not available for publication because of data licensing terms.

Wave 1 indicator values pointed to existing strengths and assets, such as 92.2% of children aged 0–5 living in the WPPN footprint having a medical home and the overwhelming majority of caregivers reporting that they read to their young children (86.9%) or encourage their older children to read (94.4%). Other indicator values showed areas of need for additional supports and were programmatic focuses for WPPN. For example, about one-quarter of young children were not engaged in an early childhood education setting. Other programmatic focuses for WPPN based on baseline indicators were kindergarten readiness and digital access (data not shown because of data licensing terms).

Out of the neighborhood survey-derived indicators, there were apparent troubling downward trends over time in two indicators of parent engagement: indicator 12, parents reading to young children (89.6% in 2018 vs. 66.6% in 2021), and indicator 14, parents talking with older children about college and career (70.2% in 2018 vs. 63.9% in 2021). The proportion of children in child care also decreased over time. We are in the process of conducting further analysis and data collection to understand the drivers of these trends to inform efforts to address them. In terms of parent engagement, national data from the National Survey of Children’s Health showed a similar trend of decreases in parents reading to their young children 2016–2019, although there was some rebound in 2020 [40]. The same survey data also showed persistent increases in parental anxiety and depression 2016–2020, which may affect parents’ ability to engage actively in their children’s educations. It is also difficult to disentangle the effects of the COVID-19 pandemic from other preexisting trends. The pandemic reinforced existing socioeconomic disparities in parental involvement in schooling through multilevel mechanisms including availability of time off or working from home, presence of one vs. two parents in the home, and access to and comfort using technology [41].

The pandemic’s effect on child care was enormous. Nationwide, two-thirds of child care centers closed in April 2020, with one-third remaining closed a year later [42]. In West Philadelphia, in the fall of 2020, child care providers were operating at reduced capacity with 56% enrollment compared to pre-pandemic enrollment numbers; by fall 2021, that number had slightly improved to 60% (data provided by Action for Early Learning, WPPN’s early childhood education partner). Reduced capacity was due to a variety of factors including COVID-19 protocols requiring fewer children in each classroom, staffing shortages causing child care programs to close classrooms, concern from parents/caregivers about sending their children back to in-person programming due to health concerns, and families experiencing challenges with obtaining subsidies, which kept them from enrolling children.

### 3.4. Communication

Data and research communication strategies evolved over the course of the grant based on stakeholder input and the evolving needs of the initiative. Communication plans were developed along with the WPPN Project Management Team, Community Advisory Council, and data-contributing partners, and products were distributed in draft form and then revised based on their input. We also provided contact information and solicited user feedback on all communication products. One outcome of this process was that rather than focusing communication on results of specific research or evaluation activities (e.g., survey results), based on stakeholder feedback, most of our communication products combined information from multiple sources and were instead organized around substantive topics identified by stakeholders as being of interest (e.g., public safety).

Figure 2 summarizes the various communication formats we used. We created interactive web dashboards with community indicators in four domains aggregated to the census-tract level for each tract within the WPPN footprint as well as citywide for comparison: demographics, education, housing, and public safety. We also developed four-page printable data briefs for each of these domains as well as easily printable versions of the graphics included in each brief. We made the dashboards, briefs, and graphics, along with information for requesting raw or aggregate indicator or survey data, available on the WPPN website [32]. We also included contact information and brief online surveys in the same area of the site for user feedback. After each neighborhood survey wave, we also created shorter, one-page snapshots summarizing survey responses aligned with WPPN initiative goals [43]. Finally, we hosted a series of community conversations in venues throughout the WPPN footprint in which we presented information on WPPN-funded activities as well as research and evaluation results and facilitated discussions with attendees on the information presented.

## 4. Discussion

We have described our approach to research and evaluation for a five-year, place-based initiative focused on supporting children and their families in a two-square-mile area of Philadelphia, Pennsylvania. Our multi-pronged evaluation approach was designed to address the multiple levels of influence on child health and development while prioritizing principles of data transparency and democratization and adhering to funder requirements.

A hallmark of place-based initiatives is that while they share some common characteristics, each is also uniquely a product of its geographic and temporal context. Accordingly, challenges we encountered over the course of the WPPN evaluation are common to evaluations of place-based initiatives but also unique in their manifestation and solutions. Much of WPPN’s evaluation structure was driven by the initiative’s status as a local initiative within the large, federally funded Promise Neighborhoods program. As articulated by Burgemeister et al. [8], policy-driven programs such as Promise Neighborhoods require finding a balance between “top–down” objectives determined by the umbrella program and “bottom–up” solutions to meet these objectives. For WPPN, “top–down” elements included the initiative’s population-level outcomes, along with the 15 indicators used to measure them and the broad data collection activities to collect this information, while the specific design of these activities was locally developed. For example, while a population-representative neighborhood survey including specific questions to measure several of the indicators was required for all Promise Neighborhood grantees, our survey’s longitudinal cohort design, sampling frame based on administrative data, community researcher survey workforce, and additional questions addressing local priorities were tailored in response to the local context in West Philadelphia.

The scope of the WPPN evaluation was also heavily driven by the five-year Promise Neighborhoods funding cycle. While this duration is typical of place-based initiative evaluations [8], it is considerably shorter than the time required to create measurable, population-level change in outcomes. After five years, a place-based initiative such as WPPN would be considered in its “middle years” according to the schematic developed by Dart et al. [30]; this is long enough to develop the foundations and systems for creating and measuring community-level change but generally not long enough for measurable population-level impact. This challenge was reflected in our evaluation design, which prioritized the creation and documentation of systems for tracking future change. We are in the process of seeking additional funding for longer-term measurement of both the WPPN grant-required outcomes (classified as intermediate outcomes in the WPPN theory of change) and the long-term outcomes described in the theory of change.

A related major challenge in designing the evaluation of WPPN was that while the grant-mandated outcomes and associated data collection activities were common across Promise Neighborhoods sites and defined beforehand, the lack of a dedicated planning period prior to the start of the grant necessitated starting evaluation activities in parallel with establishing the infrastructure and implementation planning for the initiative itself. This hampered the integration of program and evaluation planning. Particularly given the comprehensive nature of the indicators, an additional planning phase would have allowed for more careful alignment of evaluation activities with priorities and specific theorized chains of causation outlined in the theory of change.

Another common challenge is attribution [6,7,13]: because WPPN itself encompassed multiple different activities that in turn occurred along with additional investments in the community unrelated to the initiative, as well as ongoing economic and social processes unrelated to any targeted efforts, it is difficult to attribute any changes in outcomes to any specific cause or set of causes. Although there is no simple solution to this challenge, we took three steps to address it. First, we used WPPN program participation data to approximate the “dose” of WPPN exposure for students in the WPPN cohort by determining the number, type, and duration of WPPN activities in which each student participated. Under some assumptions, better outcomes among students with higher levels of exposure would suggest a causal benefit from the initiative. Second, administrative-data-based analyses of school-based outcomes will also incorporate a control group of students from schools chosen because they have similar demographics and non-WPPN-related programming to WPPN partner schools. Third, ongoing discussions between WPPN stakeholders and the collective interpretation of results help ensure that we are aware of factors outside of WPPN that may affect outcomes and take these into account when interpreting any changes in outcomes.

Another common challenge is that while fostering relationships and collaboration between stakeholders is a critical function of WPPN and other place-based initiatives for creating the conditions for sustainable community change, it is difficult to measure the impact of these efforts. The grant-mandated indicators and data collection activities did not include measures of collaboration. This was our impetus behind implementing the WPPN organizational network survey, whose primary goal was to measure these changes in relationships between organizations. Unfortunately, we were not able to conduct the first network survey until several years into the initiative, as we initially focused on funder-required data collection, limiting our ability to detect changes from baseline.

More generally, it is difficult to obtain not only measures of stakeholder collaborations but also other measures of valuable community assets. This difficulty, coupled with the preponderance of deficit-based measures among easily obtainable secondary data sources, risks creating a negatively biased view of the community that neglects existing assets. This in turn can undermine opportunities to capitalize on these assets for positive change. Publicly available data from the US Census and other sources provide easily obtainable neighborhood-level measures of economic circumstances, crime rates, and academic test scores that can highlight community needs. However, obtaining measures on locally led investments, resources, and longstanding partnerships requires time- and labor-intensive primary data collection such as resident surveys and environmental scans. Investment in the collection of these measures is important not only to create a balanced picture of community conditions but also to catalyze change.

We also encountered data- and measurement-related challenges relevant to many other place-based initiatives that share similar circumstances. Defining and estimating characteristics of our target population was difficult because of the focus on a subset of the population (i.e., children) and partial overlap between the WPPN resident and student cohorts. Uptake of the PIP by WPPN service-providing partners was hampered by slow contracting timelines that delayed the development and roll-out of the system as well as by technical capacity challenges. Because of the simultaneous initiation of implementation and evaluation activities, many providers had therefore already begun providing services without using the PIP by the time it became available, which in turn created further barriers for PIP uptake. In addition, we were not able within the timeline of the grant to resolve complex data privacy and sharing considerations that would have enabled organizations to make referrals of individual clients to each other within the system, enhancing the value of the system for providers. We also encountered administrative and legal barriers that precluded establishment of the Integrated Data System.

Finally, the COVID-19 pandemic, which spanned the second half of the WPPN grant period, had a substantial impact on both implementation and evaluation activities. Sizeable midstream changes are not uncommon in place-based initiatives. For example, the governmentally funded, multi-site Communities for Children initiative in Australia began in 2004 with a focus on children aged 0–5 years old but expanded its scope in 2009 to children aged 0–12 years [44]. Funding changes are also common [8]. However, the scale of disruption from the COVID-19 pandemic was unprecedented. From an implementation perspective, the initiative had to halt or adjust many planned funded programs and instead pivoted to activities addressing the social and economic crisis caused by the pandemic. This included intensive efforts to support virtual learning for cohort students, including increasing digital access for families, and to support food access for families. WPPN also hired a full-time COVID Resource Coordinator to help families obtain economic, health, and other supports. Research and evaluation activities also shifted. We obtained rapid funding from Drexel to develop web dashboards mapping indicators of community-level social vulnerability to crises such as the pandemic citywide [45]. Indicators were based on those used in the Center for Disease Control and Prevention’s Social Vulnerability Index [46] but adapted for local context. These dashboards eventually served as a starting point for the WPPN web dashboards. We also implemented a telephone follow-up survey of families who had previously participated in the WPPN neighborhood survey to collect information about their experiences and household needs during the pandemic. Participating families with unmet needs had the option of being contacted after the survey by the WPPN COVID Resource Coordinator for assistance. In addition to causing a shift in research and evaluation activities, the pandemic affected the availability of reporting indicator data (e.g., school attendance and state-level academic assessments). Beyond that, the widespread economic and social disruptions are a major consideration in interpretation of any of our data and render the interpretation of any trends over time particularly challenging.

Despite challenges—and in some cases as a result of grappling with challenges—there have also been notable successes of the WPPN evaluation. We have successfully built a robust infrastructure for community-engaged, participatory research and evaluation in West Philadelphia that is already being leveraged for other projects and translated for other contexts. This includes a seasoned team of faculty and professional staff who have been working with the initiative since its start. Integral to this team is a group of community researchers who are residents of West Philadelphia and who began working with WPPN as surveyors for the neighborhood survey but have since moved into other outreach, service provision, and research roles. Three of these community researchers are now employed as WPPN Data Leads charged with leading data and research communication with community stakeholders and other audiences through various formats including presentations, panel discussions, and data stories. They are also currently working with other Research and Data Core team members to co-develop specific research questions on high-priority topics that are answerable through analysis of our neighborhood survey data. Another, related key success was the development of communication products (e.g., interactive dashboards and data briefs) that have been used by a number of local organizations and agencies for program planning and grant-seeking purposes. Lastly, we developed innovative solutions to several data and measurement challenges such as using administrative data linkage for our neighborhood survey sampling frame [34], developing census-block-based population weights for estimating community indicator values despite misalignment of the WPPN footprint with US Census boundaries, and implementing a network survey to measure coalition-wide collaborations.

## 5. Conclusions

Lessons learned from WPPN research and evaluation efforts align with those from other place-based initiatives and are relevant for future initiatives. We bring the perspective of a local evaluation of an initiative nested within a multi-site, government-funded program. Furthermore, our approach may be most relevant for other initiatives in urban locations where robust community-based assets are coupled with a legacy of economic disinvestment and extractive research. Perhaps most critical, the goals of the evaluation must be clear, realistic, and transparent. Widespread population-level changes in outcomes in 5 or even 10 years may not be attainable. Instead, by building processes and a culture of using data across systems to impact health, evaluation becomes an integral part of the initiative itself as well as setting the stage for detecting and facilitating community-level changes into the future. Along the way, developing and measuring robust process-focused outcomes measures progress and holds the initiative accountable to community residents. Close collaboration between programmatic and evaluation efforts at every stage of the initiative is therefore crucial. New funding models that acknowledge the importance of these capacity-building processes, and that provide longer-term funding to allow the development and measurement of population-level outcomes responsive to systems-building approaches and in a realistic timeframe, are critical for moving forward this field and accurately determining the success of the place-based approach.

## Figures and Tables

**Table 1:** Grant-required indicators for West Philly Promise Neighborhood.

Indicator Number	Description	Specific Measure(s)	Source
1	Medical home	% children aged 0–5 who have a place where they usually go, other than an emergency room, when they are sick or in need of advice about their health	Neighborhood survey
2	Kindergarten readiness	% 3-year-old children scoring above threshold on kindergarten readiness assessment	Early childhood education provider records
		% kindergarteners scoring above threshold on kindergarten readiness assessment	School district records
		% kindergarteners receiving intensive reading support	School district records
3	Child care	% children aged <5 cared for outside of the home in a child care center or home care center	Neighborhood survey
4	Academic achievement	% students scoring above threshold on statewide Math assessment	School district records
		% students scoring above threshold on statewide English Language Arts assessment	School district records
5	School attendance	Average daily attendance	School district records
		% students absent at least 5% of school year	School district records
		% students absent at least 10% of school year	School district records
6	High school graduation	High school graduation rate	School district records
7	Career readiness	% high school graduates enrolled in college within 18 months of graduation	School district records
		% college enrollees completing college within 6 years	School district records
		% students scoring above threshold on technical education exam	School district records
8	Physical activity	% students in grades 3–12 who report engaging in 60 min of physical activity daily	School survey
9	Fruit and vegetable consumption	% students in grades 3–12 who report eating 5+ servings of fruits and vegetables daily	School survey
10	Safety	% students in grades 3–12 who report feeling safe in and traveling to school	School survey
11	Student mobility	Student mobility rate	School district records
12	Parents read to young children	% caregivers who report reading to their 0–5-year-old children 3+ times per week	Neighborhood survey
13	Parents encourage older children to read	% caregivers who report encouraging their K-8th-grade children to read outside of school	Neighborhood survey
14	Parents talk about college and career with children	% caregivers who report talking to their 9–12th-grade children about college and career	Neighborhood survey
15	Digital access	% students in grades 3–12 who report having school and home access to internet and a computer	School survey

**Table 2:** Number of West Philly Promise Neighborhood programs by focus area by academic year.

	Academic Year				
Program Focus Area	2017–2018	2018–2019	2019–2020	2020–2021	2021–2022
School Climate	4	4	8	4	4
Educational Supports (Kindergarten-12)	8	9	4	7	5
College and Career	1	2	5	5	3
Out of School Time (OST) Education	4	5	15	11	6
Community Safety/Mobility	1	1	3	2	0
Family Supports	3	3	6	5	3
Healthy Food/Nutrition	1	1	5	5	4
Early Childhood Education	2	2	2	2	2
TOTAL	24	27	48	41	27

**Table 3:** Results for West Philly Promise Neighborhood grant-required indicators derived from neighborhood survey.

Indicator Number	Description	Specific Measure	2018%	2019/2020%	2021%
1	Medical home	% children aged 0–5 who have a place where they usually go, other than an emergency room, when they are sick or in need of advice about their health	92.2	91.7	91.9
3	Child care	% children aged <5 cared for outside of the home in a child care center or home care center	74.0	68.6	56.9
12	Parents read to young children	% caregivers who report reading to their 0–5-year-old children 3+ times per week	86.9	79.1	66.6
13	Parents encourage older children to read	% caregivers who report encouraging their kingertarten-8th-grade children to read outside of school	94.4	90.6	93.1
14	Parents talk about college and career with children	% caregivers who report talking to their 9–12th-grade children about college and career	70.2	66.4	63.9

## Data Availability

WPPN neighborhood survey data can be requested here: https://westphillypn.org/request-data (accessed on 16 March 2023). Information about requesting School District of Philadelphia data can be found here: https://www.philasd.org/research/programsservices/external-research-review/requesting-data/ (accessed on 16 March 2023).
